# Machine Learning ADME Models in Practice: Four Guidelines from a Successful Lead Optimization Case Study

**DOI:** 10.1021/acsmedchemlett.4c00290

**Published:** 2024-07-25

**Authors:** Alexander S. Rich, Yvonne H. Chan, Benjamin Birnbaum, Kamran Haider, Joshua Haimson, Michael Hale, Yongxin Han, William Hickman, Klaus P. Hoeflich, Daniel Ortwine, Ayşegül Özen, David B. Belanger

**Affiliations:** ‡ Nested Therapeutics, 1030 Mass Ave, Suite 410, Cambridge, Massachusetts 02138, United States; ∥ Inductive Bio, Inc., 550 Vanderbilt Ave, #730, Brooklyn, New York 11238, United States

## Abstract

Optimization of the ADME properties and pharmacokinetic (PK) profile of compounds is one of the critical activities in any medicinal chemistry campaign to discover a future clinical candidate. Finding ways to expedite the process to address ADME/PK shortcomings and reduce the number of compounds to synthesize is highly valuable. This article provides practical guidelines and a case study on the use of ML ADME models to guide compound design in small molecule lead optimization. These guidelines highlight that ML models cannot have an impact in a vacuum: they help advance a program when they have the trust of users, are tuned to the needs of the program, and are integrated into decision-making processes in a way that complements and augments the expertise of chemists.

Optimization of ADME properties is a key challenge during the hit-to-lead and lead optimization phases of small molecule drug discovery. Machine learning (ML) models can be used to predict the outcomes of ADME-related assays such as permeability, solubility, or liver microsomal stability. They have been proposed as a tool to reduce the number of design-make-test cycles and accelerate programs.^1,2^ However, building and using ML ADME models effectively can be challenging, particularly within the context of biotech companies. Without large in-house data sets, there can be a lack of sufficient data to build performant models, particularly in the critical early stages of a program. And without the right integration of ML models into design tools, there can be a disconnect between model builders and end users, leading to limited model use.

In this Viewpoint, we share four guidelines for using ML ADME models effectively to help drive forward a drug discovery program. In describing these guidelines, we draw upon a case study of a collaboration between Nested Therapeutics and Inductive Bio. In this collaboration, Nested Therapeutics used Inductive Bio’s ADME models for lead optimization in a best-in-class program. The models were integrated into interactive tools that were used by the medicinal chemistry team, enabling rapid iteration, enhanced ideation informed by predicted data, and elevated design quality. This allowed the team to efficiently resolve permeability and metabolic stability issues, resulting in the nomination of a development candidate with excellent cell potency and cross-species PK.

## Guideline 1: Regular Time-Based and Series-Level Evaluation Gives a Realistic Picture of Model Performance and Builds Trust to Use ML Models As a Tool in the Design Process

ML model evaluation is critical to earning user trust and ensures a model is fit for use. A key step in model evaluation is selecting the subset of compounds and measurements to evaluate, which must be withheld from model training. We follow two principles when choosing these evaluation sets: they should be separated temporally from training data, and they should be stratified by program and series.

Time-based splits simulate real usage, in which a model trained on all data up to a certain date is used prospectively. This is more rigorous than the commonly used techniques of random or scaffold splitting, which can overestimate how well a model will perform due to high similarity between training and evaluation sets.^3,4^

Stratifying evaluation metrics by program and series is important because ML models can vary in their performance across projects and chemotypes, in a way that can be hard to predict *a priori*.^5^ Proactively measuring performance at project and series levels informs project teams on where and for what purpose models can be confidently used.

In our collaboration, we used these two principles to build initial trust in the ML models and to continually validate the models’ fitness for use. We performed a time-based split using data from an existing program that had completed lead optimization, evaluating performance across three distinct chemical series, to confirm the general suitability of the ML models to Nested’s data. We then performed an additional time-based evaluation on existing early data from the program of interest. From there, the ML models were used on an ongoing basis, and performance was re-evaluated weekly using a time-based split (see Guideline 3). We reported metrics for the project as a whole and by series where appropriate according to definitions crafted by the project team.

## Guideline 2: Training on a Combination of “Global” Curated Data and “Local” Program Data Leads to the Best Model Performance

When developing ML ADME models for a project team, one can create a local model trained solely on the program’s measured data, as in traditional QSAR approaches.^6^ Alternatively, one might use a global model that has already been built using large external data sets to predict a given property.^7,8^ An approach that balances these extremes is to train a model that combines nonproject global data with data from the project itself. This can be done by simply including all available data when training a model^2,9,10^ or by using other more sophisticated fine-tuning approaches.^5^ Studies have found that fine-tuned models trained with combined local and global data perform better than those trained with local or global data alone.^5,9^

The models used in our collaboration followed this best practice of combining global and local data for training. The models were initially developed using a curated global proprietary data set and were then fine-tuned by adding project data. Training was performed using a graph neural network model architecture.^11^

To explore how the fine-tuned global model compared to local-only or global-only alternatives, we analyzed performance of the Inductive models against versions of the models trained only on external curated data (global-only) and models trained locally using a QSAR software tool implementing AutoML (local-only).^12^ For each of human liver microsomal stability (HLM), rat liver microsomal stability (RLM), Madin-Darby Canine Kidney (MDCK) permeability (MDCK AB), and MDCK efflux ratio (MDCK ER), we created a temporal evaluation split by choosing the first 100 compounds measured as local training data and the next 100 compounds as a test set.

**Figure 1:**
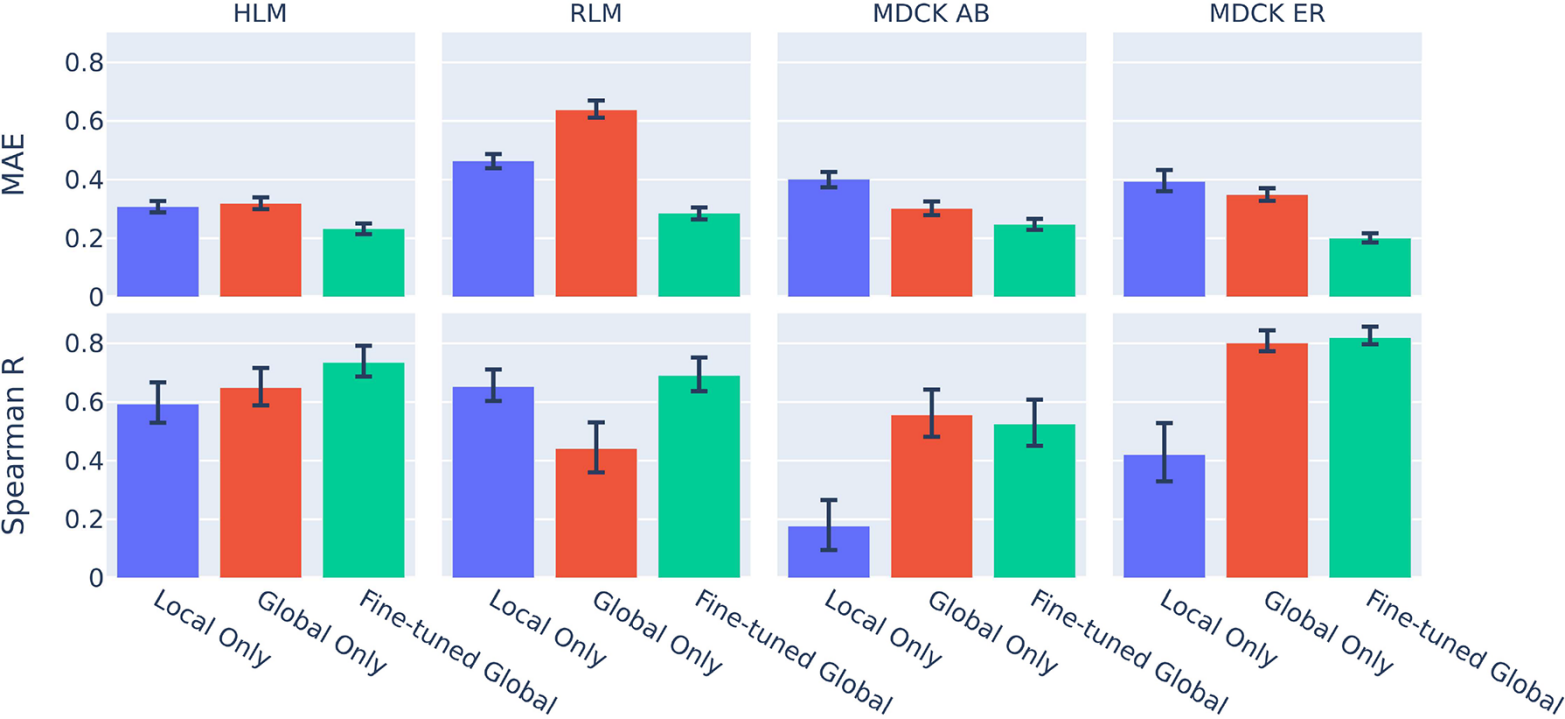
Performance of models trained on program data only (local only), nonprogram data only (global only), and on combined data (fine-tuned global), on temporally split test sets for HLM, RLM, MDCK AB, and MDCK ER. Error bars represent 68% bootstrapped confidence intervals. MAE (mean absolute error) units are in log 10 (mL/min/kg) for HLM and RLM, log 10 (μcm/s) for MDCK AB, and log 10 (ratio) for ER.

As seen in Figure 1, the fine-tuned global modeling approach generally performed best across the assays. It achieved the lowest MAE (Mean Absolute Error) across all four properties, and the highest Spearman rank correlation across all assays except MDCK AB, where the global model correlation was slightly higher.

In this program, the global training seemed most helpful in predicting the MDCK measurements, and it was least helpful in RLM. This cannot be easily explained in terms of similarity of test compounds to those in the global training sets. For all four assays, no more than 2% of the Nested compounds had compounds in the global training set with a Tanimoto similarity of >0.3 (calculated with Morgan fingerprints, radius 2). However, we observed a surprising divergence in measured HLM and RLM intrinsic clearance in the Nested compounds, with RLM compounds exhibiting a median 8 times higher clearance. This difference between species was not anticipated by the global model, which predicted roughly equal clearance (leading to a high MAE for RLM), but was captured by the fine-tuned global model. This emphasizes the value of validating models on early program data, as well as that of training on local data.

## Guideline 3: Frequent Model Retraining Enables ML Models to Learn Local SAR As a Program Shifts into New Chemical Space and Encounters Activity Cliffs

Throughout lead optimization, new experimental data are collected that may improve model performance. Tested compounds may also move into new chemical space, potentially worsening performance.^13^ These factors create an incentive for frequent model retraining so the models can learn the local SAR from the experimental data and maintain accuracy.^14,15^

Monthly retraining has been shown to provide a boost in performance compared to less frequent schedules across a variety of ADME end points.^16^ Frequent retraining can be particularly useful for rapidly adjusting to activity cliffs.^14,17^ Weekly retraining has been reported to be beneficial^2^ and aligns well with the weekly cycle of design meetings common in drug programs. While weekly retraining may sometimes incorporate only a few new compounds, it also strengthens user trust by ensuring that model predictions are always informed by the most recent and relevant assay readouts.

Throughout the course of our collaboration, we applied weekly retraining to keep our microsomal stability and permeability models up to date. A retrospective analysis confirms that frequent training aided performance for HLM stability, the property for which data was collected most consistently throughout the time course of the program. When we split HLM measurements into periods of 1 month and evaluated predictions from the model deployed at the beginning of that month, we observed an average Spearman R of 0.65. If we instead used the model deployed 1 month previous, the Spearman R fell to 0.55. An additional month lag dropped it further to 0.49.

We also observed the helpfulness of model retraining for adjusting to activity cliffs. At one point, a change to a substitution position in a ring was discovered to cause a surprising several-fold jump in microsomal clearance. While the model did not predict this jump in advance, retraining weekly allowed it to rapidly adjust to the observed data and begin making appropriate predictions for additional compounds with the new motif.

## Guideline 4: To Maximize Impact on the Design Process, ML Models Should Be Interactive, Interpretable, and Integrated with Other Tools

The best ML ADME model will not have an impact unless it is actively used.^1^ We have found that models have the best chance of being used effectively if they are *integrated*, *interactive*, and *interpretable*. *Integrated* models are available within software tools that computational and medicinal chemists are already using to guide decision-making. *Interactive* models provide real-time predictions as a chemist ideates new designs, rather than working only via bulk scoring or with a long computation time. And finally, *interpretable* models provide not only a predicted assay value but additional relevant information such as atom-level visualizations indicating important regions of the molecule for a given property. These three principles were brought together to make the Nested–Inductive collaboration successful.

At the start of the collaboration, the program was in early lead optimization with the goal of demonstrating in vivo target engagement at a projected low human dose. Compound 1 (Table 1) showed moderate cellular activity, good permeability, and HLM stability, yet in vitro/in vivo dog and rat clearance needed improvement.

**Table 1:** Key Compounds from the Case Study Campaign and Their Properties

compound #	target engagement assay (nM)	HLM T1/2 (min)	RLM T1/2 (min)	dog LM T1/2 (min)	MDCK Papp (ER)	projected human dose
1	752	83	37	2	13.8 (0.8)	
2	100	82	44	22	3.6 (2.6)	
3	263	82	32	13	4.7 (2.2)	
4	137	65	65	57	8.1 (0.9)	4× higher than desired
5	124	83	72	60	7.4 (0.8)	desired

To address these goals, the computational team at Nested vetted and deployed docking, strain energy prediction, and atomistic simulations including free energy predictions. Frequently retrained ML ADME models from Inductive were integrated into the existing computational infrastructure via an application programming interface (API). This allowed ADME predictions to be presented in the context of complementary modeling output, reducing the number of compounds requiring evaluation through computationally intensive approaches before selection for synthesis.

**Figure 2:**
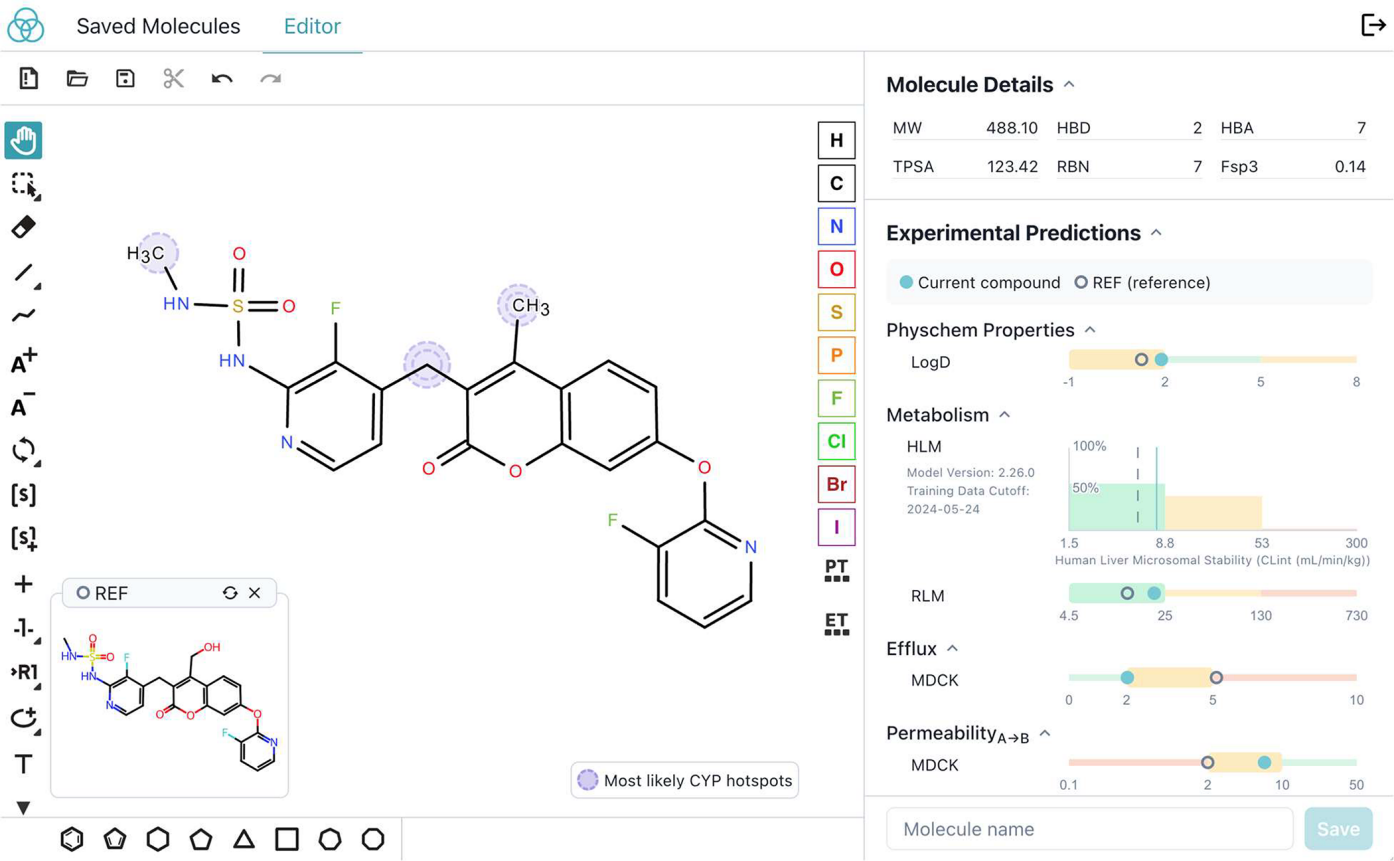
A screenshot of the interactive design environment with ML ADME predictions, comparison to a reference compound, and highlights of sites of likely metabolism.

As understanding of the SAR of the shallow, flexible target improved, chemists made increasing use of the interactive ML application (Figure 2). In contrast to a batch scoring interface, the interactive application enabled chemists to rapidly draw ideas, get instant feedback, and iterate to find the most promising candidates for synthesis. This interactive approach allowed scientists to explore and reason about the SAR of multiple design steps at once rather than primarily considering single point design changes. The interpretability tools, including reactivity-based prediction of likely sites of metabolism,^18^ further helped guide design changes.

Ultimately, the ML ADME models of permeability and metabolic stability were used in tandem with an existing structure-based design workflow to elevate the overall quality of the designs. Using this strategy, Nested co-optimized two regions of the compound to identify potent, permeable compounds **2** and **3**. The team then used the ML application to fine-tune physiochemical properties and address metabolic soft-spots without sacrificing permeability or potency. Compound **4** advanced to cross-species pharmacokinetics (PK) and was soon followed, within a few weeks, by the identification of compound **5** with exquisite cell potency and cross-species PK.

## Conclusion

By following these guidelines, ML ADME models can be made an integral part of the lead optimization process within biotech. Using ML models does not negate the necessity of collecting experimental ADME data or eliminate the risk of synthesizing compounds with unfavorable properties. Nonetheless, it can meaningfully accelerate a program and improve a chemist’s ability to cooperatively optimize PK and potency to achieve a desired PD outcome. When an ADME challenge arises for a particular property, an effective ML model can be used to quickly generate and evaluate ideas that might fix the problem, which can then be experimentally validated. When ADME properties are not a current challenge, ML models can help maintain good properties while tuning other attributes and flag potential issues. Brought together, these uses help turn a lead into a development candidate faster and identify solutions to property tradeoffs that enable compounds with better PK–PD profiles.
